# Stool consistency is significantly associated with pain perception

**DOI:** 10.1371/journal.pone.0182859

**Published:** 2017-08-09

**Authors:** Yukiko Shiro, Young-Chang Arai, Tatsunori Ikemoto, Kazuhiro Hayashi

**Affiliations:** 1 Department of Physical Therapy, Faculty of Rehabilitation Sciences, Nagoya Gakuin University, Nagoya, Japan; 2 Multidisciplinary Pain Center, Aichi Medical University, Nagakute, Japan; 3 Institute of Physical Fitness, Sports Medicine and Rehabilitation, School of Medicine, Aichi Medical University, Nagakute, Japan; 4 Institute of Rehabilitation, Aichi Medical University Hospital, Nagakute, Japan; University Hospital Llandough, UNITED KINGDOM

## Abstract

**Background:**

Commensal as well as pathogenic bacteria can influence a variety of gut functions, thereby leading to constipation and diarrhea in severe cases. In fact, several researchers have reported evidence supporting the association between stool consistency or constipation and the Gut microbiome (GM) composition and dysbiosis. GM influences the human health and disease via the gut-brain axis. We thus hypothesized that the pathogenic bacteria increases pain perception to some extent, which means that there could be an association between stool consistency or constipation and pain perception of healthy subjects.

**Design:**

Observational study.

**Objectives:**

The aim of the present study was to investigate the association between stool consistency or constipation and pain perception of healthy subjects.

**Methods:**

Thirty-eight healthy subjects participated in this study. The participants were assessed on their usual stool form (the Bristol Stool Form Scale: BSFS), constipation (the Cleveland Clinic Constipation score: CCS), degree of obesity, pain perception by mechanical stimulus, cold pain threshold, and a questionnaire on psychological state.

**Results:**

The BSFS was significantly and positively associated with pain perception, and showed a significant association with anxiety states. Furthermore, pain perception was significantly associated with anxiety states. However, there were no significant associations between the CCS and any independent variables. In addition, we found that a significant predictor to the pain perception was BSFS. Moreover, there were significant relationships among the psychological states, BSFS and obesity.

**Conclusion:**

These results suggest that the stool form is associated with pain perception and anxiety status.

## Introduction

Commensal as well as pathogenic bacteria have been known to influence a variety of gut functions for a long time, thereby leading to constipation and diarrhea in severe cases. Also, the human microbiome has been recognized as a substantial contributor to human nutrition, health and disease in the past decade [1]. Accumulating clinical and animal research-based evidence has made us realize the significance of gut microbiome (GM) to the healthy and homeostatic operation throughout the whole host body. The GM may contribute to the regulation of multiple neuro-chemical and neuro-metabolic pathways through a complex series of highly interactive and symbiotic host-microbiome signaling systems that interconnect several organs such as the gastrointestinal tract and skin with the central nervous system (CNS), thereby influencing host neuromodulatory, neurotransmission and neuroimmune functions [2, 3]. Therefore, we can imagine that imbalance of the normal GM and dysbiosis has been linked not only with host gastrointestinal conditions such as irritable bowel syndrome (IBS) and obesity but also with host neuropsychiatric diseases such as anxiety and schizophrenia.

Pain can become intractable when pathophysiological factors such as neural injury and inflammation are processed in a maladaptive way by psychosocial factors. A process of learning and sensitization of pain involves activity-dependent changes (that is, plasticity) at many levels from the molecular to the network level because several neuromodulators indispensably make the nervous system more sensitive to painful stimulation and increase and strengthen pain perception, thereby ending up neuroplasticity of pain perception [4, 5]. We thus imagine that the GM could increase pain perception to some extent.

Commensal as well as pathogenic bacteria can influence a variety of gut functions possibly mediated by bacterial metabolites or bacterial cell components or through interactions between bacterial cells and the animal host cells, thereby leading to constipation and diarrhea in bad cases. In fact, several researchers have reported evidence supporting an association between stool consistency and the Gut microbiome (GM) composition and dysbiosis. [6, 7]. Since we hypothesized that pathogenic bacteria affects pain perception, the aim of the present study was to investigate the association between stool consistency and pain perception.

## Methods

After receiving approval from the Nagoya Gakuin University Board of Ethics (reference number: 2016–27) and obtaining written informed consent, 38 healthy subjects (24men and 14 women, 22.5±1.1 years) were recruited for the present study. The exclusion criteria included serious conditions such as neurological disease (e.g., spinal disc herniation, stroke, and hereditary disease), diabetes, menstruation, or administration of sedatives, analgesics, or other medication.

The participants were assessed on their usual stool form, constipation, degree of obesity, pain sensation by mechanical stimulus, cold pain threshold, and a questionnaire on their psychological states.

Stool form was assessed using the Bristol Stool Form Scale (BSFS). The BSFS is an ordinal scale of stool types ranging from the hardest (Type 1) to the softest (Type 7) with pictorial representations of each stool type. Types 1 and 2 are considered to be abnormally hard stools (and in conjunction with other symptoms indicative of constipation) while Types 6 and 7 are considered abnormally liquid stools (and in conjunction with other symptoms indicative of diarrhea). Types 3, 4 and 5 are generally considered to be the most normal stool forms [8]. Constipation was rated using the Cleveland Clinic Constipation score (CCS). The CCS is calculated based on eight factors. These factors consist of frequency of bowel movement, painful evacuation, incomplete evacuation, abdominal pain, length of time per attempt, assistance for evacuation, unsuccessful attempts at evacuation per 24 hours and duration of constipation. Scores for each factor ranged from 0 to 4 (with the exception of ‘assistance for defecation,’ which was 0 to 2) [9, 10]. Degree of obesity was assessed by using the body mass index (BMI).

Pain sensation by mechanical stimuli was evaluated by using the self-made von Frey monofilament (VFM). The diameters of VFMs were 1.5 mm in all filaments and the length of each monofilament (GCK-60^®^ Mitsubishi Reyon Co. Ltd., Japan) was adjusted to produce a different force (100g and 600g). Each painful stimulus was given to 2 points on an inter-digital site (600g to second-third and 100g to fourth-fifth) on the rights hand for 5 seconds [11, 12]. The intensity of pain for each stimulus was rated using the visual analogue scale (pain-VAS) where 0mm indicated no pain and 100mm the greatest pain possible [11, 12]. Cold pain threshold (CPT) was assessed by having participants immerse their left hands up to the wrist in cold water as far as possible for up to 2 minutes. The cold water was maintained up to 10±1°C [13]. We measured the time elapsed from the moment subjects immersed their hands in the cold water up until the time they first felt pain and removed their hands. Pain assessments were performed in 60-s intervals for each stimulus in a measurement.

Psychological state was assessed by the Hospital Anxiety and Depression Scale (HADS), the Pain Catastrophizing Scale (PCS) and the State-Trait Anxiety Inventory Questionnaire (STAI). HADS is a self-report screening instrument for negative moods. HADS consists of 14 items; the anxiety (HADS-A) and depression (HADS-D) subscales each include 7 items. Again, higher scores (zero to 21 points for anxiety and depression alike) indicate greater degrees of anxiety and depression [14]. PCS consists of 13 items, and subjects rate how frequently they have catastrophizing about pain, with higher scores (zero to 52 points) indicating greater degrees of catastrophizing. PCS is composed of three subscales: rumination (e.g., “I keep thinking about how much it hurts”), magnification (e.g., “I wonder whether something serious may happen”), and helplessness (e.g., “There is nothing I can do to reduce the intensity of the pain”) [15]. STAI consists of 40 statements across two subscales; a State anxiety subscale (STAI-S, items 1–20), and a Trait anxiety subscale (STAI-T, items 1–20). To score, the 20 responses for each scale are tallied and the total score for both ranges from 20 to 80, with a lower score reflecting a better psychological state [16].

### Data analyses

First, we used Gpower software to determine the sample size for this study. The sample size required a minimum of 34 subjects to show an effect size of 0.4 with a significant level of 0.05 (α = 0.05) and a power of 80% (β = 0.20). All data were analyzed using SPSS Version 20 (IBM, New York, USA). Data were presented as median and range because each assigned sample resulted in not only a parametric but also a non-parametric distribution. There were 2 steps to our linear regression analyses. In the first step, we analyzed the relationship between the pain-VAS (100g, 600g), the CPT or psychological state (HADS, PCS, STAI) and independent variables (i.e. BFS, CCS, BMI) using Spearman’s correlation coefficients (ρ). In the second step, a stepwise multiple linear regression analysis was performed to predict the pain-VAS, the CPT or psychological state of the independent variables. A p-value <0.05 was considered statistically significant.

## Results

Participant characteristics are presented in Table 1. The BSFS was significantly and positively associated with pain-VAS 100g (Table 2). In terms of psychological state, the BSFS showed a significant association with STAI. BMI showed a significant association with HADS-D and STAI. Furthermore, pain-VASs displayed a significant positive association with STAI (Table 2). However, there were no significant associations between the CCS and any independent variable. In addition, we found that significant predictors to the pain-VAS 100g were BSFS and PCS, while a predictor to the pain-VAS 600g was BSFS (Table 3). Moreover, there were significant relationships between HADS-D and BMI, STAI and BSFS, and STAI and BMI (Table 4).

**Table 1:** Participant characteristics.

	n = 38
Women (%)	36.8
Age (yr)	22 (21–25)
BMI (kg/m^2^)	21.1 (17.4–31.6)
BSFS	4 (1–6)
CCS	3 (0–12)
pain-VAS 100g (mm)	10 (0–28)
Pain-VAS 600g (mm)	55 (10–70)
CPT (sec)	23.3 (6.4–83.4)
HADS-A	7 (0–16)
HADS-D	5 (0–15)
PCS	15 (0–52)
STAI-S	47 (17–59)
STAI-T	47 (22–57)

Value: median (range)

BMI: Body Mass Index, BSFS: Bristol Stool Form Scale, CCS: Cleveland Clinic Constipation score, CPT: Cold Pain Threshold, HADS: Hospital Anxiety and Depression Scale (A; Anxiety, D; Depression), PCS: Pain Catastrophizing Scale, STAI: State-Trait Anxiety Inventory.

**Table 2:** A simple linear regression analysis.

	BSFS	CCS	BMI	HADS-A	HADS-D	PCS	STAI-S	STAI-T
pain-VAS_100g_	0.341*	0.055	0.190	0.196	0.245	0.318	0.357*	0.386*
pain-VAS_600g_	0.268	0.053	0.075	0.098	0.049	0.307	0.360*	0.260
CPT	-0.154	-0.181	-0.008	-0.030	0.051	-0.001	0.053	0.025
HADS-A	-0.027	0.157	0.288	-	-	-	-	-
HADS-D	0.166	-0.149	0.487*	-	-	-	-	-
PCS	-0.041	0.143	0.049	-	-	-	-	-
STAI-S	0.480**	-0.193	0.395*	-	-	-	-	-
STAI-T	0.500**	-0.043	0.398*	-	-	-	-	-

Value: correlation coefficient,

*, **: *p* < 0.05, 0.01

BSFS: Bristol Stool Form Scale, CCS: Cleveland Clinic Constipation score, BMI: Body Mass Index, CPT: Cold Pain Threshold, HADS: Hospital Anxiety and Depression Scale (A; Anxiety, D; Depression), PCS: Pain Catastrophizing Scale, STAI: State-Trait Anxiety Inventory (S: State anxiety, T: Trait anxiety)

**Table 3:** Multiple linear regression analysis with pain sensation as a dependent variable.

	Variables	Adjusted R^2^	B	β	*p* value	95% CI for B	
						Lower limit	Upper limit
pain-VAS_100g_		0.251					
	Constant		-2.230		0.570	-10.117	8.569
	BSFS		2.929	0.427	0005	0.949	4.909
	PCS		0.181	0.327	0.028	0.021	0.431
pain-VAS_600g_		0.109					
	Constant		25.277		0.018	4.623	45.931
	BSFS		6.366	0.365	0.024	0.876	11.855
CPT	none						

Partial regression coefficient: B, Standard partial regression: β

CPT: Cold Pain Threshold, BSFS: Bristol Stool Form Scale, PCS: Pain Catastrophizing Scale

**Table 4:** Multiple linear regression analysis with the psychological scale as a dependent variable.

	Variables	Adjusted R^2^	B	β	*p* value	95% CI for B	
						Lower limit	Upper limit
HADS-A	none						
HADS-D		0.146					
	Constant		-6.352		0.147	-15.054	2.349
	BMI		5.190	0.411	0.010	0.130	0.908
PCS	none						
STAI-S		0.361					
	Constant		8.550		0.372	-10.639	27.739
	BSFS		4.929	0.534	0.000	2.455	7.403
	BMI		0.851	0.287	0.037	0.057	1.646
STAI-T		0.421					
	Constant		10.320		0.230	-6.839	27.478
	BSFS		5.156	0.594	0.000	2.944	7.368
	BMI		0.741	0.266	0.041	0.031	1.452

Partial regression coefficient: B, Standard partial regression: β

HADS: Hospital Anxiety and Depression Scale (A; Anxiety, D; Depression), PCS: Pain Catastrophizing Scale, STAI: State-Trait Anxiety Inventory (S: State anxiety, T: Trait anxiety), BMI: Body Mass Index, BSFS: Bristol Stool Form Scale

## Discussion

This study showed that the stool form was associated with pain perception and anxiety status. Especially, the higher pain sensitivity and anxiety status were, the looser and more watery their stool was. On the other hand, there were no significant correlations between constipation and pain perception or psychological status.

In the past decade, the human microbiome has been recognized as a contributor to the health status of the host body. The GM may contribute to the regulation of multiple neuro-chemical and neuro-metabolic pathways. On the other hand, pain is a multidimensional experience that incorporates nociceptive, affective, and cognitive networks [17] and thus pain can become intractable when pathophysiological factors are processed in a maladaptive way by psychosocial factors. That is, most of patients with intractable pain experience physical and psychological stress, and psychologically impaired states (e.g., depression, anxiety). A process of learning and sensitization of pain involves activity-dependent changes (that is, plasticity) at many levels from the molecular to the network level because several neuromodulators indispensably increase and strengthen pain perception, thereby ending up neuroplasticity of pain perception [4, 5]. In addition, risk factors for pain include obesity [18], and there is a relationship between obesity and higher levels of pain intensity [19]. Our present study showed that there was an association between pain sensation and anxiety states. BMI showed a relationship with the psychological states, but was not related to pain perception. However, interestingly, our results revealed that there were some relationships between pain perception or psychological states and the BSFS score. The BSFS score is known to be associated with GM composition, dysbiosis, and this score is negatively correlated with gut species richness [6, 7].

Current studies focus on describing the relationship between the GM and disease states (e.g., obesity-related disease, liver disease, colorectal cancer and IBS) [20]. For example, disregulation of host responses secondary to dysbiosis within the gut lumen could affect distant anatomical sites through activation of host immune responses. This may be a mechanism in rheumatoid arthritis [21]. In another study, by fecal transplantation from hypertensive human donors to germ-free mice, the recipient mice exhibited significantly higher blood pressure as compared to controls. This result showed that the hypertension might be affected by the GM [22].

Furthermore, the GM appears to influence the development of emotional behavior, stress- and pain-modulation systems, and brain neurotransmitter systems [3]. Previous studies in animal models have suggested behavioral changes following manipulation of the GM, including effects on behavior associated with stress [23], anxiety [24], and depression [25]. Naseribafrouei et al. observed a general underrepresentation of the Bacteroidetes phylum in depressed patients [26]. In addition, in several studies, researchers observed that the probiotic bacteria influenced the pain perception during colorectal distension [27, 28].

A bidirectional communication between the brain and gut exists, which is referred to as the gut-brain axis. There are many potential direct and indirect pathways through which the GM can modulate the gut-brain axis. They include neurotransmission (e.g., catecholamine, serotonin, dynorphin, and cytokines) and neuronal pathway (e.g., vagus and enteric nervous system) [29, 30]. For example, approximately 95% of serotonin in the human body is contained within the gut [31]. Peripheral serotonin is involved in the regulation of pain perception [32], mood and cognition [33]. These studies supported the neuronal control of the gut-brain axis transits between the CNS and enteric nervous system via autonomic- and peripheral-nervous systems [30]. This has implications for pain perception. For example, IBS is a functional gastrointestinal disorder that is characterized by chronic or recurrent abdominal pain associated with abnormal bowel movements. As mentioned above, IBS also may be affected by neurotransmissions (e.g. serotonin, catecholamine, endocannabinoid, and cytokines) [34]. Besides, there is a tendency for IBS patients to harbour a higher average count of Bacteroidetes compared to healthy controls [32]. Furthermore, abdominal pain was positively correlated with Bacteroidetes in IBS patients [35]. In addition, the Bacteroidetes is positively associated with the BSFS score [7]. Our results showed that the BSFS score was positively correlated with pain sensitization and psychologically low states. Thus, the microbiota dysbiosis might increase pain perception and anxiety states not only in IBS but also in healthy subjects.

There were several limitations to the present study because this study included elements of a qualitative study. First, we did not measure GM and short chain fatty acids. There is emerging evidence that changes in microbiota diversity lead to variations in short chain fatty acids [36]. Secondly, this study was conducted on young healthy subjects. We need further evaluation for the relation between GM and pain perception in older adults and chronic pain patients.

In conclusion, the present study showed that the stool form was correlated with pain perception and anxiety status. Especially, abnormally liquid stool was more related to pain sensitization and anxiety status than hard stool. These findings indicate that the microbiota dysbiosis might be involved in pain sensitization and psychologically low states. Thus, our results suggest that assessing stool form in patients with chronic pain is important.

## Ethical approval

Receiving approval from the Nagoya Gakuin University Board of Ethics (reference number: 2016–27).

## Supporting information

S1 File The data are provided as its supporting information. (XLSX)
